# The heritability of spatial memory and caching behaviour in a food-storing bird

**DOI:** 10.1007/s10071-025-01950-5

**Published:** 2025-04-10

**Authors:** Tas I.F. Vámos, Ella McCallum, Rachael C. Shaw

**Affiliations:** School of Biological Sciences, Victoria University of Wellington, Wellington, New Zealand

**Keywords:** Bayesian animal model, Petroica longipes, Cognitive evolution, Cognitive ecology, Quantitative genetics, Cognitive plasticity

## Abstract

Research examining how cognitive traits evolve in the wild has focussed on finding evidence of the ‘Darwinian holy trinity’– consistent individual variation in cognitive performance that is linked to fitness and has a heritable component. In food-storing birds, there is growing evidence of selection for more accurate spatial memory performance. However, for selection to act on variation in spatial memory performance, it must also have a genetic component. In this study, we used Bayesian animal models to evaluate the heritability of memory performance in a spatial reference memory task in a population of wild toutouwai (North Island robin, *Petroica longipes*). We also estimated the heritability of variation in measures of the caching behaviour that spatial memory theoretically underpins. We found little evidence of heritability in either spatial memory performance or caching measures, as credible intervals were large with lower bounds close to zero. This result could suggest that individual variation in memory performance and caching behaviour may be primarily due to non-genetic factors. For example, variation in toutouwai spatial memory could be shaped largely by the cognitive demands of altering caching decisions in response to cache theft risk. In this scenario, the underlying mechanisms determining and linking spatial memory and caching behaviour would need to be reconsidered. Alternatively, the large credible intervals for our heritability estimates may be an artefact of small sample size. Therefore, to progress our understanding of how cognition evolves, it is crucial to establish long-term studies in the wild to collect cognitive performance data from as many individuals as possible over successive generations, with the goal of increasing the reliability of heritability estimates.

**Supplementary Information:**

The online version contains supplementary material available at 10.1007/s10071-025-01950-5.

## Introduction

Cognition encompasses the variety of processes through which animals perceive, process, store, and use information (Shettleworth 2010). In the past decade, several studies have shown that consistent individual differences in cognitive performance are associated with differential reproduction and/or survival (Ashton et al. 2018; Rochais et al. 2023; Soravia et al. 2022; Vámos and Shaw 2022; Welklin et al. 2024). However, for cognitive evolution to occur via natural selection, it is insufficient for cognitive trait variation to be linked to fitness; it must also be heritable (Morand-Ferron and Quinn 2015). To date, there has been far less research on the heritability of cognitive traits in natural populations of animals, with the existing studies providing mixed results regarding the roles of genetics and environmental variables in shaping individual cognitive variation within populations (Branch et al. 2022; Dall et al. 2012; Morand-Ferron and Quinn 2015; Speechley et al. 2024). Understanding the extent to which cognitive traits are heritable is therefore crucial for furthering our understanding of the nature of cognitive evolution (Croston et al. 2015).

Spatial memory is one of the most intensively studied cognitive traits, as the ability to retain and use spatial information is a basic requirement for any animal that moves between two or more locations (Gibson and Kamil 2009; Paul et al. 2009). Studies on humans suggest a heritable component to spatial memory ability and associated brain structure (Blokland et al. 2011) and activity (McGee 1979), while captive studies on non-human animals have yielded a diversity of findings, ranging from strong evidence for heritability in spatial memory task performance in chimpanzees (*Pan troglodytes*) (Hopkins et al. 2014) to limited or no heritability in the spatial learning abilities of delicate skinks (*Lampripholis delicata*) (Vardi et al. 2020). Findings of little to no heritability for spatial memory performance may indicate that individual cognitive variation is due to environmental factors (Pravosudov et al. 2015), or neural plasticity (Sherry and Hoshooley 2010). In the case of the latter, an especially relevant case study is that of London taxi drivers, who exhibit a significant increase in hippocampal grey matter mass during their training (Woollett and Maguire 2011).

Animals that store food for later retrieval (i.e. caching species) represent valuable model systems for examining the heritability of spatial memory in ecologically relevant contexts (Croston et al. 2015). For caching species, having an accurate spatial memory is vital; the inability to remember where food is stored could lead to poorer body condition, reduced reproductive potential, or even death (Kamil and Gould 2008; Sherry 1984). Classic comparative research revealed that food-storing species outperform non-caching species in tasks involving the recall of spatial information (Clayton and Krebs 1994; Krebs et al. 1990). Moreover, research comparing populations of a caching species, the mountain chickadee (*Poecile gambeli*), revealed that birds from harsher climates, where recalling cache locations is more crucial for survival due to increased energetic requirements, also had enlarged hippocampal structures (Pravosudov and Clayton 2002). These findings were among some of the first to support the long-held theory that selection has shaped the spatial cognitive abilities of food-storing birds to aid them in creating and recovering caches (Sherry et al. 1988). More recently, this adaptive specialisation hypothesis has been given further support by research in toutouwai (North Island robin, *Petroica longipes*) (Shaw et al. 2019) and mountain chickadees (Sonnenberg et al. 2019; Welklin et al. 2024), revealing that individual differences in performance on spatial memory tasks can be associated with differential reproductive success (Shaw et al. 2019) and survival (Sonnenberg et al. 2019; Welklin et al. 2024). Additionally, a genome-wide association study using 42 wild mountain chickadees revealed around 10 loci that accounted for 87% of phenotypic variance in spatial cognitive performance, suggesting moderate to high heritability of spatial cognitive phenotypes in the population (Branch et al. 2022).

Cognitive traits become visible to selection when they underpin behaviours that are closely linked to survival and reproduction (Real 1993). In the case of caching species, individual variation in caching behaviour is the assumed behavioural target for selection on spatial cognitive abilities (reviewed in Vámos and Shaw 2022). Empirically evaluating the link between spatial memory and caching behaviour in the wild is difficult, as the large spatial and temporal scales over which many species cache make direct quantification of caching impractical (Cristol 2001; Vander Wall and Balda 1977). Consistent individual variation in detailed measures of caching behaviour has recently been documented in wild toutouwai (Vámos and Shaw 2024), and has been linked to variation in spatial memory performance, with better spatial reference memory performance linked to a propensity for caching at greater distances from a food source (Vámos and Shaw 2025). However, the heritability of this individual variation in caching behaviour has not yet been examined. In fact, to date there has been no research on the heritability of variation in caching behaviour in any caching species, despite its assumed role as a behavioural target for selection on underlying spatial memory abilities.

The toutouwai provides an ideal model system for evaluating the heritability of spatial memory performance and caching behaviour in a natural population. The toutouwai is a small passerine species endemic to the North Island of New Zealand. Toutouwai create caches of whole or dismembered invertebrate prey in their small territories, which are typically retrieved within several hours to several days of caching (Higgins and Peter 2002). Being largely fearless towards humans, toutouwai are easily observed during caching and can be readily trained to interact with experimental apparatuses, allowing detailed quantification of both caching behaviour and spatial memory performance (Alexander et al. 2005; Shaw et al. 2015). As a result, it is possible to evaluate both individual differences in cognition, as well as the putative behavioural target for selection (Shaw et al. 2019; Vámos and Shaw 2022). Toutouwai are largely socially and genetically monogamous (Ardern et al. 1997), reside in the same territories year-round (McGavin 2009), and offspring typically do not disperse far from their natal territories (Higgins and Peter 2002). These factors allow detailed and reliable pedigrees to be constructed on populations that are monitored over the course of many years (McCallum and Shaw 2023).

In this study, we evaluate the heritability of spatial memory performance and individual variation in caching behaviour using our long-term study population of wild toutouwai at Zealandia Te Māra a Tāne ecosanctuary in Wellington, New Zealand. We use Bayesian animal models (de Villemereuil 2024; Hadfield 2010) to create pedigree-based heritability estimates, which have previously been used to assess heritability in toutouwai inhibitory control performance (McCallum and Shaw 2023). To quantify individual spatial memory performance, we measured a bird’s performance on a spatial reference memory task in which it was required to remember one consistently rewarded location out of eight possible options across repeated trials. For caching behaviour, we use data from our previously published research on the repeatability of caching behaviour, in which we quantified the distance that birds travelled to cache provided food items, as well as the number of unique cache sites used (Vámos and Shaw 2024). A study comparing individual variation in spatial reference memory performance with our measures of individual variation in caching behaviour found a relationship between memory performance and the distance at which items were cached (Vámos and Shaw 2025). Under the adaptive specialisation hypothesis (Sherry et al. 1988), we predicted that there would be evidence for heritability in both spatial memory performance and caching behaviour, as this would indicate that there is a genetic component to spatial memory and caching behaviour that is available for selection to act on. By contrast, finding no evidence for heritability in either trait could indicate that variation in toutouwai spatial cognition may be primarily non-genetic and have limited selective potential. Finally, evidence of heritability in caching behaviour but not spatial memory performance (or vice versa) could indicate that there are unexplained sources of heritability and/or variation that have not been taken into account by the adaptive specialisation hypothesis (Sherry et al. 1988), requiring a re-evaluation of the relationship between spatial cognition and caching behaviour.

## Methods

### Subjects and study site

The subjects were 71 (N_males_ = 38, N_females_ = 33) wild toutouwai residing in Zealandia Te Māra a Tāne ecosanctuary. The birds inhabit a study area of approximately 25 ha consisting of regenerating native forest and introduced pine forest. Toutouwai are territorial, with birds in our study population residing year-round in small territories of < 1 ha (Shaw et al. 2019). For the experiments described below, we tested each bird in isolation on their winter territory. Since 2014, the birds within this study area have been monitored and banded with individually identifiable coloured band combinations (Shaw et al. 2015). As this population has been continuously monitored for more than a decade, many birds in our population were banded as nestlings in the study area and recruited into the breeding population as adults. We therefore had a knowledge of parent-offspring relationships and were able to construct a detailed pedigree of the relationships between known birds in our population. No molecular studies of extra-pair paternity have been conducted in our population, however, previous research has found that toutouwai are socially and genetically monogamous (Ardern et al. 1997). We therefore assumed that our pedigree based on social parent-offspring relationships would also represent the genetic maternal and paternal relationships between the birds of our study population.

### Ethical note

The study was carried out under permit from the Department of Conservation 56493-FAU and with the approval of the Victoria University of Wellington Animal Ethics Committee (application number 26824).

### Quantifying individual variation in spatial reference memory performance

**Fig. 1:**
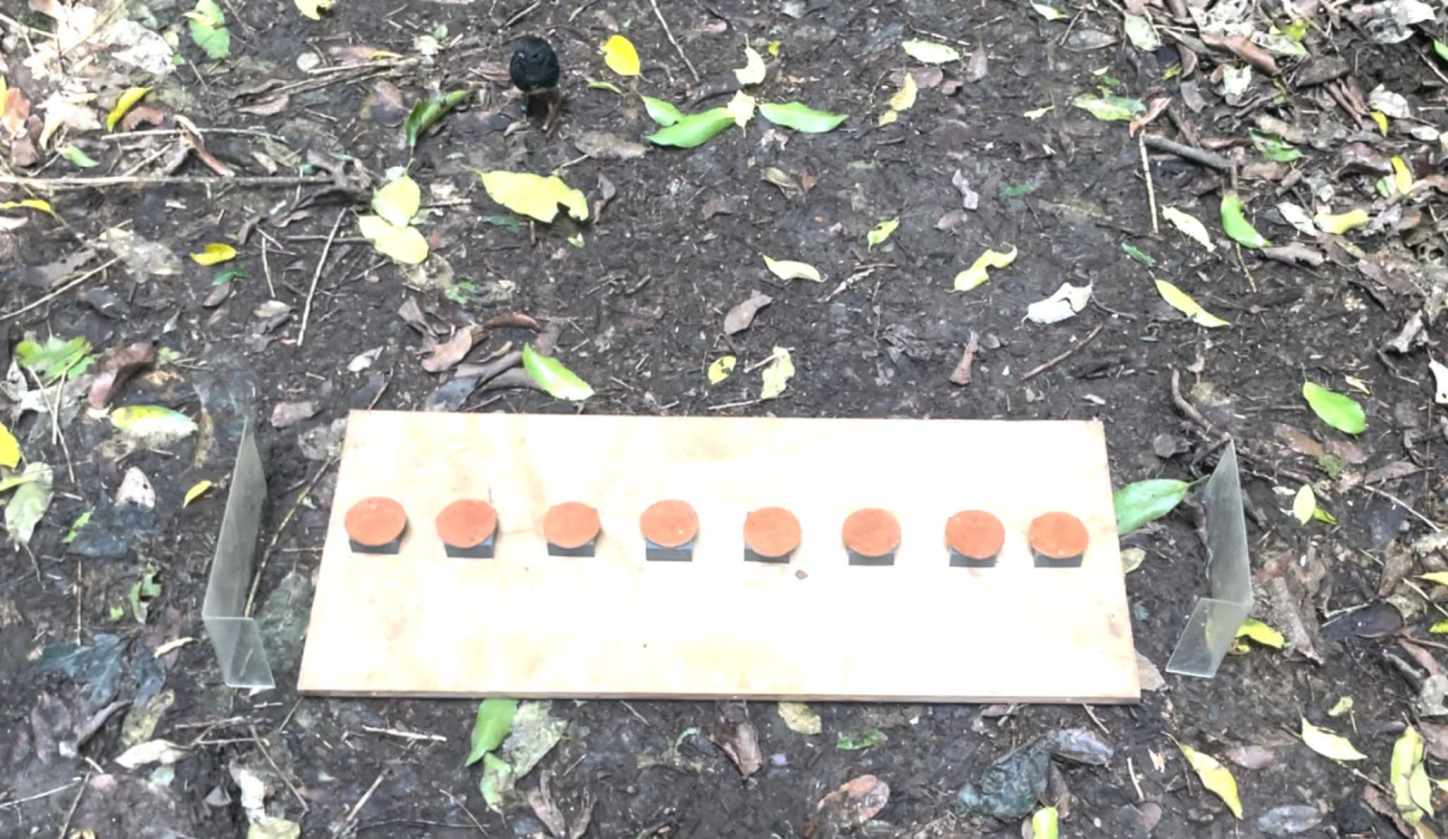
A bird participating in the spatial reference memory task. Photo credit: T.V

To assess the heritability of spatial memory performance, we quantified individual performance in spatial reference memory, which is defined as memory for spatial information that remains reliable over time (McNamara 2013), We tested 40 (N_males_ = 22, N_females_ = 18) individuals during the austral winter of July– August 2021. Further details on the testing protocol are available in Vámos and Shaw (2025). We used a spatial discrimination task in which a bird had to learn one rewarded location out of eight possible locations. We presented a bird with a linear array of 8 possible reward locations, which were spaced 5 cm apart on a wooden board (85 × 30 × 1 cm) (Fig. 1). Each reward location consisted of a square plastic tile (4 × 4 × 2 cm) with a well drilled in the centre (2 cm diameter, 1 cm depth), covered by a leather lid (5 cm diameter).

Before the task began, a bird was trained to flip the leather lid using a shaping procedure consisting of three levels. In the first level, the lid was leaning against the tile but not obstructing access to a mealworm (*Tenebrio molitor* larvae) reward. In the second level, the lid partially concealed the well so that the mealworm was visible but not accessible unless the lid was removed. In the final level, the lid completely covered the mealworm. To pass a lid flipping training level the bird had to retrieve the mealworm reward in three consecutive trials within 2 min. If a bird failed a trial, it regressed to the previous level and had to pass again to continue.

During the task, the third tile from the end of the array always contained a single, freshly killed mealworm. The side of the array that was rewarded was randomly assigned and counterbalanced among birds, and remained constant across all of a bird’s trials. Two transparent barriers were placed at the ends of the array to prevent a bird approaching the array from either end. Each bird received an initial probe trial in which it could flip all lids in the array to discover that only one location was rewarded. All birds flipped all lids during this probe trial. Immediately following the probe trial, we gave a bird its first session of 14 test trials. The following day we gave the bird a second and final session of 16 trials, so that each bird had 30 test trials in total. Test trials began as soon as the experimenter took up position, by sitting approximately 1 m behind the board. During a trial, a bird was allowed to flip as many lids as required to locate the mealworm reward. Immediately after retrieving the reward, the apparatus was covered with a towel for 30 s. During this intertrial interval, the tiles were randomly shuffled to prevent the bird using subtle visual cues, the rewarded position was re-filled, and all lids were replaced. The towel was then removed and the bird allowed to approach for its next trial. If a bird did not interact with the apparatus within 2 min, the trial was re-set. If a bird failed to interact with the task within 2 min in three consecutive trials, the session ended and was re-started on the following testing day, as this suggests a lack of motivation.

The final test trial (trial 30) in the task was unrewarded, to control for the possibility of the bird using olfactory cues to locate the reward. If birds had been relying on the smell of the reward to locate the position, then their performance should have regressed to chance at this point. This did not occur, indicating that birds were relying on spatial reference memory; see Vámos and Shaw 2025. All birds we attempted to test completed the task. All trials were filmed using an iPhone 6. Examples of task trials are given in Online Resource 1.

For our heritability analyses, we defined the spatial reference memory performance measure as the total cumulative lids that a bird flipped across its 30 test trials. Fewer lid flips indicated less error, and thus better performance (i.e. a perfectly scoring bird would achieve a score of 30 by flipping the correct lid on each trial, while the worst possible score a bird could achieve was 240).

### Quantifying individual variation in caching behaviour

To assess the heritability of caching behaviour– the presumed behavioural target of selection for spatial reference memory in caching birds (Vámos and Shaw 2022)– we used data that had been collected in a study of the repeatability of toutouwai caching decisions (Vámos and Shaw 2024). Data were collected from 69 (N_males_ = 37, N_females_ = 32) individuals across two years, during the austral autumns and winters (April– August) of 2020 and 2021. Twenty-six (N_males_ = 14, N_females_ = 12) of these individuals were tested in both years. We gave a bird three caching sessions each year, with one per day on three consecutive testing days. If inclement weather prevented fieldwork, testing resumed on the next possible day. During a caching session, we identified a central location on a bird’s winter territory where we gave a bird one freshly killed mealworm at a time, placed on a wooden board. After the bird either ate or cached the provided mealworm, the experimenter immediately placed a new mealworm in the same location. This process continued until the bird had cached five mealworms in total during the session. Therefore, each bird cached a standardised 15 mealworms across its three caching sessions in a year. There were minor differences between sessions run in 2020 and 2021. In 2020, between each session the feeding location was moved 5 m, while in 2021 the task occurred concurrently with an inhibitory control task and the testing location did not move (McCallum and Shaw 2023; Vámos and Shaw 2024). These differences across years are accounted for in our heritability analyses by including year as a factor in all models using data from both years. All instances of caching were filmed using an iPhone 6. Examples of caching instances are given in Online Resource 2.

For heritability analyses, we used two measures of caching behaviour: site number and caching distance. Site number was the total number of unique cache sites that a bird used during the three caching sessions in each year. Caching distance was the mean distance (m) a bird travelled from the food source to each cache site for the 15 caches it made during its three caching sessions in each year. This was measured as a straight-line distance from feeding location to cache site using a tape measure (distances ≤5 m) or Bushnell rangefinder (distances > 5 m). We only quantified instances of caching in which the bird was seen to deposit a mealworm in a cache site; the rare cases where the final location of the mealworm was not seen (4.9% of caching instances) were not included in analyses (Vámos and Shaw 2024). We have previously shown that both site number and caching distance are repeatable measures across years and thus represent an individual’s caching behaviour independent of short-term environmental effects (Vámos and Shaw 2024). Using more unique sites suggests that a bird has a propensity to scatter caches as opposed to clumping all items in a few sites, while a larger mean distance travelled between cache sites and the food source suggests that an individual preferentially disperses caches sites on their territory, which increases the energy/time invested to create each cache.

### Heritability analyses

All analyses were conducted using R v. 4.2.1 (R Core Team 2022). We ran three separate Bayesian animal models using the package ‘MCMCglmm’ (Hadfield 2010) to estimate the heritability of spatial reference memory performance, the heritability of the propensity to scatter caches (measured as the total unique cache sites used in a caching session) and the heritability of the propensity to disperse caches (measured as the mean distance in m between cache sites and the food source). Pedigree information (see supplementary material) was obtained from monitoring records without genetic analysis, as toutouwai are thought to be largely socially and genetically monogamous (Ardern et al. 1997). We also included birds of unknown parentage in the pedigree (i.e., immigrants and birds hatched prior to 2014 when monitoring began), as animal models take into account all types of relatedness (de Villemereuil et al. 2013). The average kinship coefficient for all individuals tested was 0.009, with the kinship coefficient for each pair of individuals representing the probability that two randomly sampled alleles at the same autosomal locus are identical by descent (Jiang et al. 2022).

Bayesian animal models for spatial reference memory performance and total cache sites were run as Poisson generalised linear mixed models, and mean cache site distance was run as a Gaussian linear mixed model. As random effects, all models included individual ID linked to the pedigree, and a dominance matrix calculated using the ‘nadiv’ package (Wolak 2012) to account for genetic dominance effects, which can add to non-additive genetic variance (de Villemereuil 2024). We also included testing year as a fixed factor in models for total cache sites and mean cache site distance to control for differences in caching behaviour between years (e.g., due to minor variation in the test protocol or environmental conditions; see above and Vámos and Shaw 2024 for details). Finally, models for total cache sites and mean cache site distance included individual ID independent of the pedigree as a random factor to account for permanent environment effects (environmental influences on individual phenotype that are stable across repeated measures (Kruuk 2004). It was necessary to fit permanent environment effects for these two models as 26 individuals took part in the caching experiments in both years, and the models would otherwise treat these repeated measures as arising from clones (de Villemereuil 2024).

Models were run using a weakly informative, parameter expanded prior (V = 1, nu = 1, alpha.mu = 0, alpha. V = 1000). We ran models for 500,000 iterations, with a burn-in period of 50,000 iterations and a thinning interval of 100 iterations. For each model, the additive genetic variance was divided by the total phenotypic variance to estimate h^2^ (de Villemereuil 2024).

## Results

Individual spatial reference memory performance (measured as the total number of locations that an individual searched to locate rewards across 30 trials) ranged from 72 to 133 (Mean ± s.d. = 98.8 ± 13.0). The total number of cache sites that toutouwai used to cache 15 mealworms during their three caching sessions ranged from 1 to 13 (Mean ± s.d. = 5.5 ± 2.3), and the mean distance travelled from the food source to a cache site ranged from 2.3 to 8.7 m (Mean ± s.d. = 5.2 ± 1.3 m). More in-depth analysis and discussion of these results are available in two previous articles (Vámos and Shaw 2024, 2025).

**Fig. 2:**
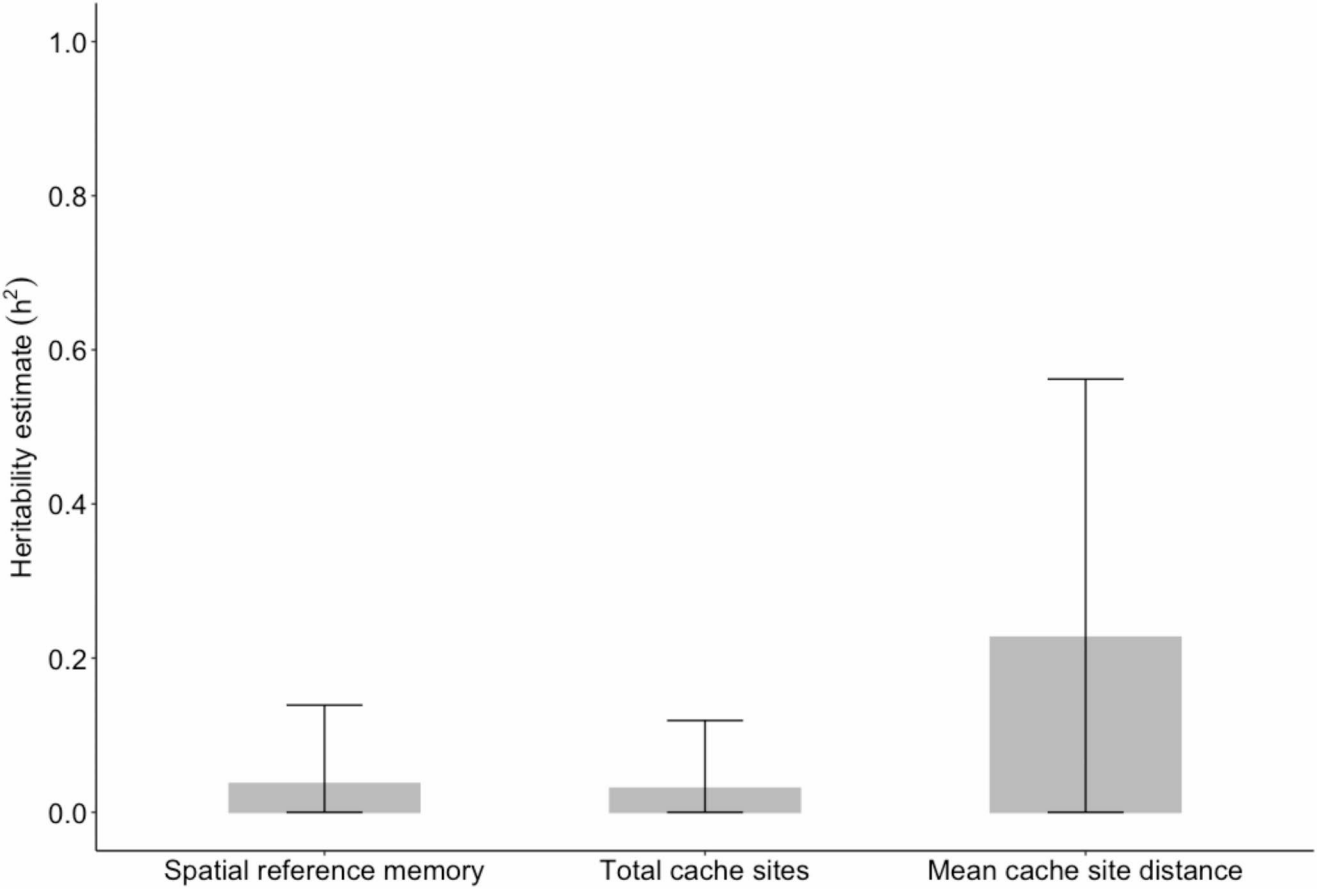
Heritability estimates (h^2^) for spatial reference memory performance, the total number of unique cache sites used (a measure of cache scattering propensity), and mean distance travelled from the food source to each cache site (a measure of cache dispersion propensity). Error bars show 95% credible intervals for heritability estimates

Heritability estimates were negligible for spatial reference memory performance (h^2^ = 0.037, 95% CI = 0.000–0.139) and total cache sites used (h^2^ = 0.031, 95% CI = 0.000–0.119), and low for mean cache site distance (h^2^ = 0.227, 95% CI = 0.000–0.562) (Fig. 2). The 95% credible intervals indicated high uncertainty surrounding all heritability estimates, and the lower bounds were very close to zero (de Villemereuil 2024).

## Discussion

In this study, we used Bayesian animal models to evaluate the heritability of individual performance on a spatial reference memory task, as well as the heritability of two measures of caching behaviour in wild toutouwai. Contrary to our expectations under the adaptive specialisation hypothesis for the evolution of spatial memory in caching species (Sherry et al. 1988), we found little evidence for heritability in either spatial memory performance or individual caching preferences. Our results reinforce previous research that found a lack of heritability in toutouwai cognitive performance (McCallum and Shaw 2023), but contrast with recent research on food-storing chickadees that suggests a genetic component to spatial memory performance (Branch et al. 2022).

The lack of evidence for heritability in toutouwai spatial reference memory performance may suggest that toutouwai spatial memory is highly plastic. Seasonal neural plasticity is well-established in migratory (Barkan et al. 2017), brood-parasitic (Clayton et al. 1997), and food-storing birds (Sherry and Hoshooley 2010). In the latter example, black-capped chickadees (*Poecile atricapillus*), experience an increase in neurogenesis and the size of the hippocampus during the autumn and winter, potentially in response to the increased spatial memory demands associated with caching (Sherry and Hoshooley 2010). Thus food-storing birds’ memory abilities can be plastic and could be influenced by individual experience. However, while many avian caching species in the Northern hemisphere experience large climatic variation across seasons and display strong caching seasonality, toutouwai in our population inhabit a relatively stable climate and cache year-round (although caching is reduced during the breeding season when offspring are being provisioned). Therefore, rather than plasticity in toutouwai memory performance being the result of strong seasonal variation in caching behaviour, an individual’s experience with cache theft may play a larger role. When the risk of pilferage is high, individual toutouwai tend to create more, more widely-scattered cache sites (Burns and Steer 2006; Van Horik and Burns 2007). Scattering items across more sites and dispersing these caches sites more widely may place a greater cognitive load on an individual’s spatial memory (Tello-Ramos et al. 2019). Thus, if some individuals experience more cache theft (e.g. because they are unable to physically defend caches or have a large population of heterospecific pilferers in their territory) and respond by investing more in scattering and dispersing caches, then they may also develop consistently better spatial memory capabilities. This phenomenon would be analogous to the example of London cab drivers developing advanced spatial abilities for navigating city streets during their training (Woollett and Maguire 2011).

We have shown in a previous study that individual variation in cache placement decisions (i.e. the number of sites used and the distance travelled to cache) is repeatable over the long-term (Vámos and Shaw 2024), and that caching distance is correlated with spatial reference memory performance, with birds that placed caches further from the food source having better spatial reference memory performance (Vámos and Shaw 2025). As repeatability reflects the upper limit for heritability (Boake 1989), we expected that these results could indicate a heritable component to caching behaviour. Yet our analyses revealed negligible heritability for the number of cache sites used and low heritability for the mean distance travelled to cache, with large 95% credible intervals. Thus, it appears likely caching decisions in toutouwai may be considerably plastic. An individual’s decisions about where to create caches may be shaped by their past experiences with cache theft, with birds experiencing theft more likely to scatter numerous caches, whereas a bird that experiences no such loss (or is able to prevent theft via guarding) may tend to clump caches closer together (Vámos and Shaw 2024). In this study we only investigated the heritability of two caching measures previously found to be repeatable in toutouwai; other aspects of caching behaviour that we did not measure here, such as preference for certain types of cache sites or rates of retrieval, may be more heritable.

The size of the credible intervals for our heritability estimates potentially points to a general issue with using animal models to estimate the heritability of complex behaviours and cognitive performance in the wild. A recent study quantifying heritability in inhibitory control performance in great tits (*Parus major*) employed an animal model approach similar to ours (Thompson et al. 2024). Their heritability estimates for the number of errors and latency to escape during an inhibitory control task were similar to our low estimate for the distance travelled to cache. However, 95% credible intervals for both heritability estimates were wide with the lower bound stated as 0.00, despite having a sample size of almost 400 individuals and a detailed pedigree. The same study also included a genome wide association approach which found several SNPs that were linked to the number of errors during the inhibitory control task and accounted for 21% of variance in task performance. This latter evidence for a heritable component to inhibitory control performance, despite the large uncertainty of the animal model-based estimate, could suggest that animal model-based approaches may rarely yield heritability estimates with precise credible intervals when used in the context of cognitive studies in the wild.

Our results present an interesting contrast with recent findings from a genome-wide association study on another caching passerine, the mountain chickadee, which revealed covariance between performance in a spatial reference memory task that was similar to the task used in our study and genomic variation (Branch et al. 2022). We can only speculate as to the reason for the apparent differences in the heritability of spatial memory performance between these two species. Mountain chickadees and toutouwai are distantly related taxa that each evolved caching independently, occupy differing climatic regions (strongly seasonal vs. mild temperate) and cache different food types (seeds vs. invertebrates) over very different timescales (weeks or months vs. hours or days). Chickadees can also create hundreds of cache sites (Croston et al. 2016), whereas toutouwai cache less frequently in fewer sites (Powlesland 1981; Vámos and Shaw 2024). The relative importance of genetic and environmental determinants of individual variation could therefore differ substantially between the two species. Selection may be comparatively relaxed in toutouwai, with the costs of forgetting/losing caches being less risky given that large invertebrate prey are available even in the coldest times of year in Wellington (Vergara et al. 2020), whereas a chickadee in an alpine area may rely to a much greater extent on cached food during the coldest and/or snowiest periods of winter. However, it is also possible that spatial memory and caching behaviour in toutouwai have been under strong directional selection in the past, which can erode genetic variation and result in low heritability estimates (Falconer and Mackay 1996; Mousseau and Roff 1987). Additionally, work on the heritability of cognitive traits in mountain chickadees has focused on identifying genetic loci that correlate with spatial memory performance (Branch et al. 2022), as opposed to the pedigree-based heritability analysis we employ here, making direct comparisons challenging.

Comparative laboratory-based research on storing and non-storing species supports the adaptive specialisation hypothesis for the evolution of spatial memory abilities (Brodbeck 1994; Clayton and Krebs 1994; Krebs et al. 1990; McGregor and Healy 1999). Yet the lack of evidence for heritability in toutouwai spatial memory performance in the current study appears inconsistent with the hypothesis that spatial memory is under selection in this species. There are a variety of challenges involved in working with wild animals to quantify individual cognitive and behavioural differences (highlighted in Rowe and Healy 2014). We cannot discount the possibility that behaviour during our spatial memory and caching experiments was influenced by variables other than the target traits. If these confounding variables have little or no heritability, this could have masked any low-level heritability that exists in toutouwai spatial reference memory performance and caching behaviour. Nonetheless, we were able to control for many potential confounding variables either during testing or in our analyses, such as avoiding the presence of predators or conspecifics during testing, or ensuring that body condition did not affect our behavioural and cognitive measures (see Vámos and Shaw 2024 for details).

Our relatively small sample size and an incomplete pedigree reduced the power of our analyses (Dingemanse et al. 2002; McCallum and Shaw 2023; Quinn et al. 2006; Speechley et al. 2024; Vardi et al. 2020). Moreover, because of our limited sample size, we did not attempt to control for maternal and paternal effects (non-additive genetic parental effects on offspring phenotype) as 31 of our 71 study subjects were of unknown parentage, and our dataset contained few half-siblings due to high levels of pair-bond retention across years (Wilson et al. 2010). A solution to this in future toutouwai research may be to simplify the spatial reference memory task protocol and present it to as many individuals as possible and over a longer period. However, such broader, long-term studies come with logistical and funding difficulties, as highlighted in a recent review of the importance of long-term studies in studying evolution (Stroud and Ratcliff 2025). As our study population continues to be monitored through time, a more complete pedigree will likely allow for more precise estimations of heritability in the future. Finally, previous research has shown that apparently minor changes in the scale and context of cognitive tasks can produce markedly different performances by individuals (Troisi et al. 2021). Changing aspects of our spatial reference memory task to make it more accurately reflect the scale on which caching occurs (e.g., by having a much larger array) could result in individual performance scores that that are more reflective of the cognitive abilities employed by toutouwai during caching. However, given that our caching and spatial reference memory scores are linked (Vámos and Shaw 2025), we consider it unlikely that our choice of task is solely to blame for the apparent lack of heritability.

In summary, we examined the heritability of spatial reference memory performance in a wild, food-storing bird, as well as the heritability of caching behaviour, which is the presumed to be one of the primary behavioural targets for selection on spatial memory in this species. Our findings of low or absent heritability in both memory performance and caching behaviour could arise from our limited sample size and an incomplete pedigree. Alternatively, the lack of evidence for heritability in these traits may suggest that spatial memory performance and caching behaviour in food-storing species such as toutouwai are considerably more plastic and responsive to the environmental conditions within a bird’s lifetime than is predicted under the adaptive specialisation hypothesis. This second possibility calls for a re-evaluation of the causal relationship between spatial memory and caching behaviour. Rather than an individual’s spatial cognitive abilities influencing caching behaviour, the circumstances under which caching occurs may ‘train’ an individual’s spatial cognitive abilities. Future research should focus on validating the results of this study with a larger sample size and also address the non-heritable causes of variation in caching and its underlying cognition, with particular focus on examining the influence of pilferage risk on the shaping of individual differences in spatial cognition.

## Electronic supplementary material

Below is the link to the electronic supplementary material.

Supplementary Material 1

Supplementary Material 2

## Data Availability

All data and code are available at: https://figshare.com/s/5fe35af841f490e3f64a, https://figshare.com/s/953c7a3bafe8b4676996, https://figshare.com/s/f6e03763624b1c6fa10a.
